# S100B Mitigates Cytoskeletal and Mitochondrial Alterations in a Glial Cell Model of Autosomal Recessive Spastic Ataxia of Charlevoix-Saguenay

**DOI:** 10.1007/s12035-025-05057-3

**Published:** 2025-05-19

**Authors:** Ana Sofia Boasinha, Fernanda Murtinheira, Susana Solá, Cláudio M. Gomes, Federico Herrera

**Affiliations:** 1 BioISI – Instituto de Biosistemas e Ciências integrativas, Faculdade de Ciências da Universidade de Lisboa, 1749-016 Lisbon, Portugal; 2 Departamento de Química E Bioquímica, Faculdade de Ciências, Universidade de Lisboa, 1749-016 Lisboa, Portugal; 3 Research Institute for Medicines (iMed.ULisboa), Faculty of Pharmacy, Universidade de Lisboa, 1649-003 Lisbon, Portugal; 4 Faculdade de Ciências (Edificio C8), Universidade de Lisboa, Rua Ernesto de Vasconcelos, 1600-548 Lisboa, Portugal

**Keywords:** Molecular chaperones, ARSACS, Sacsin, S100B, Mitochondria, Intermediate filaments, Astroglia

## Abstract

Autosomal Recessive Spastic Ataxia of Charlevoix-Saguenay (ARSACS) is an early-onset neurological disorder caused by mutations in the *SACS* gene, resulting in the loss of sacsin function. Sacsin is a multidomain protein that plays key roles in chaperone regulation, protein quality control, and neurofilament dynamics. Sacsin deficiency leads to disruption of intermediate filament and mitochondrial networks. S100B, a multifunctional brain-enriched protein, exhibits protective neuroprotective functions that include chaperone activity and interactions with filament proteins and mitochondria. In this study, we used an established astroglial C6 cell model of ARSACS to investigate the potential compensatory effects of S100B on sacsin loss with respect to neurofilament integrity and mitochondrial morphological and functional hallmarks. Our results demonstrate that sacsin deletion induces S100B upregulation at both mRNA and protein levels, with the S100B protein colocalizing with perinuclear nestin aggregates and filamentous mitochondria networks. Genetic silencing and pharmacological inhibition of S100B exacerbate filament protein aggregation and mitochondrial defects, while supplementation with exogenous recombinant S100B improves ARSACS hallmarks, including decreased nestin aggregates. These findings provide evidence for functional compensation of sacsin loss by S100B in glial cells, and suggests a potential role for glial cells in ARSACS.

**Supplementary Information:**

The online version contains supplementary material available at 10.1007/s12035-025-05057-3.

## Introduction

Autosomal recessive spastic ataxia of Charlevoix-Saguenay (ARSACS) is an early-onset disease characterized by cerebellar ataxia, peripheral neuropathy, and nystagmus. Although the exact prevalence of ARSACS is unknown, it is considered one of the more common recessive ataxias worldwide [1–3]. Mutations in the SACS gene, located on chromosome 13, lead to loss of sacsin function and development of ARSACS [4]. Although the physiological role of sacsin remains unknown, several studies suggest a role as a molecular chaperone, consistent with its domain composition which includes a J domain, homologous to the DnaJ chaperone (Hsp40) [5–7].

Cell models of ARSACS reveal that sacsin loss-of-function disrupts intermediate filament (IF) [6, 8, 9] and mitochondrial networks [10–12], and this could be related to sacsin´s chaperone activity [5, 8, 11]. Re-expression of sacsin´s J domain in Sacsin-knockout cells effectively resolves abnormal filament bundles, underscoring sacsin role as a chaperone in maintaining IF assembly and homeostasis [13]. Proteostasis modulators such as HSP70 are relocalized and enriched in disrupted IF networks, suggesting a compensatory response to maintain filament proteostasis [9]. Additionally, sacsin localizes to mitochondria and may be implicated in chaperone-assisted selective mitophagy following mitochondrial damage [5, 10]

Proteostasis defects and energy metabolism dysfunction trigger cellular stress responses, often resulting in the release of factors associated with neuroinflammation, a hallmark of many neurodegenerative diseases, including ARSACS [14]. This response includes the activation of microglia and astrocytes and the expression and release of inflammatory cytokines and alarmins, such as IL-6, IL-1β, TNF-α and S100B [15]. S100B is one of the most abundant proteins in the brain, mainly expressed by astrocytes but also neurons and other non-neuronal cell types in the central nervous system [16]. Increased S100B levels have been found in Alzheimer's and Parkinson’s diseases, multiple sclerosis, Down's syndrome, schizophrenia, Tourette's syndrome, epilepsy, and migraine [17]. S100B has been shown to function as a molecular chaperone, co-localizing with senile plaques in AD and delaying the onset of Aβ42 aggregation [18]. S100B can have both neuroprotective and toxic effects on CNS cells, generally depending on the concentration [16, 17, 19].

Protective S100B functions include molecular chaperone activity countering the aggregation, toxicity and cell to cell spreading of Tau and Aβ42, which is mediated by regulatory interactions direct interaction with these proteins [20–22]. Besides Tau, S100B interacts with other elements of the cytoskeleton [16], such as desmin, GFAP and Vimentin intermediate filaments [20]; and colocalizes with microtubules and centrosomes [21, 23]. S100B also interacts with membrane organelles such as mitochondria, where it displays positive and negative effects on mitochondrial function in a concentration-dependent manner [24, 25]. Interestingly, pharmacological inhibition of S100B leads to mitochondrial dysfunction [26, 27].

These pieces of evidence suggest a potential role for S100B as a housekeeping chaperone involved in the assembly and maintenance of intermediate filaments and mitochondrial function, two functions that could be relevant in the context of ARSACS. To test this hypothesis, we employed an astroglial model of ARSACS to evaluate the possible compensatory effects of S100B on the intermediate filament and mitochondrial morphological hallmarks caused by sacsin loss.

## Materials and Methods

### Reagents

Dulbecco’s modified Eagle’s medium (DMEM) low glucose was purchased from Biowest (Nuaillé, France). Fetal bovine serum (FBS) was acquired from Sigma-Aldrich (St. Louis, MO, USA). Penicillin–Streptomycin solution, Bradford Dye Reagent, Pierce ECL Plus Western Blotting Substrate, Hoechst 33,342, rabbit anti-vimentin polyclonal antibody, horseradish peroxidase-conjugated secondary antibodies—goat anti-rabbit IgG (H + L) and goat anti-mouse IgG (H + L)—and goat anti mouse or anti-rabbit secondary antibodies conjugated to AlexaFluor 488, AlexaFluor 546 or AlexaFluor 647 fluorophores were purchased from Thermo Fisher Scientific (Waltham, USA). MitoView Fix 640 was acquired from Biotium (California, USA). Protease Inhibitor Cocktail EDTA Free and rabbit anti-S100B was purchased from Abcam (Cambridge, United Kingdom). PhosSTOP tablets (phosphatase inhibitor cocktail) were purchased from Roche (Basel, Switzerland). Mouse anti-Sacsin (N-terminal), anti-GAPDH, and anti-Nestin antibodies were purchased from Santa Cruz Biotechnology Inc (Dallas, TX, USA). Mouse anti-S100B and rabbit Anti-Sacsin (C-terminal) antibodies, as well as 3-(4,5-Dimethylthiazol-2-yl)−2,5 diphenyltetrazolium bromide (DMSO) were purchased from Merck Millipore-Sigma™ (St. Louis, MO, USA). S100B_cerulean_pcDNA3.1(+) plasmid was synthesized by GenScript Biotech (New Jersey, USA). S100B siRNA (mouse) and the control siRNA-A were purchased from Santa Cruz Biotechnology Inc (Dallas, TX, USA). Pentamidine isethionate salt was also purchased from Santa Cruz Biotechnology Inc (Dallas, TX, USA). Human S100B was expressed in *Escherichia coli* cells and purified as previously described [28]. Lactate dehydrogenase (LDH) activity and total ATP levels were determined by means of the LDH Cytotoxicity Detection Kit (Takara Bio, Cat. No. MK401, Shiga, Japan) and the ATP Determination Kit (Invitrogen, Thermo Fisher Scientific, Cat. No. A22066, Waltham, MA, USA), respectively, according to the manufacturer’s instructions. Absorbance and luminescence were read using a VICTOR3 plate reader (PerkinElmer, United States).

### Cell Culture and Treatments

C6 rat glioblastoma cells were acquired from ATCC (CCL-107 TM). A sacsin knockout C6 cell line (C6^Sacs−/−^) was previously produced by means of a CRISPR/Cas9 approach [8]. Both cell lines were grown to confluence in DMEM, supplemented with 10% (v/v) FBS, 1% (v/v) Penicillin/Streptomycin and maintained at 37 °C in a humidified atmosphere containing 5% CO_2_. Cell lines were periodically tested for mycoplasma contamination by means of a commercial test (Biontex Laboratories, Munich, Germany). For experiments, cells were plated on sterile plastic dishes or on sterile glass coverslips and allowed to adhere for 16–24 h before experiments and/or sample preparation. For experiments involving 24-h incubation periods, cells were seeded in 35 mm dishes at density of 5 × 10^5^ cells/dish, in 24-well plates at a density of 5 × 10^4^ cells/well and in 96-well plates at a density of 5 × 10^3^ cells/well. For experiments involving 48-h incubation periods, cells were seeded with half of the density. For experiments with pentamidine, C6^Sacs−/−^ cells were treated at a final drug concentration of 0.5 µM for 48 h.

### Cell Transfection

Transfections were done 24-h after seeding by means of the jetPRIME transfection reagent (DNA:transfection reagent 1:3). For S100B overexpression, a plasmid containing this protein fused with the cerulean fluorescent protein was transfected into both C6 and C6^Sacs−/−^ cells. In 35 mm plates, 0.5 and 0.25 μg of DNA were used for C6 and C6^Sacs−/−^ cells, respectively. In 24-well plates, half of the DNA was used for each cell line. Cells were incubated with the transfection mixture for 24 h, the medium was then changed, and cells were grown for 24 additional hours before sample preparation. Inhibition of S100B protein expression using siRNA (2 μg) was done with the jetPRIME transfection reagent following a similar protocol. The sequence of S100B siRNA is a pool of three different siRNA duplexes including siRNA1: 5′- GAA CAU GAG UGA GAU UAG ATT 3′ (sense), 5′- UCU AAU CUC ACU CAU GUU CTT-3′ (antisense); siRNA2: 5′-GAG ACG GUC AUG CAA GAA ATT-3′ (sense), 5′-UUU CUU GCA UGA CCG UCU CTT-3′ (antisense); and siRNA3: 5′- GAG UCA GGU CUC AGU GAU ATT-3′ (sense), 5′-UAU CAC UGA GAC CUG ACU CTT3′ (antisense). A mock control was performed using a commercial siRNA scrambled sequence.

### Western Blot

Western blot was performed as previously described [8]. Briefly, cells were lysed with native lysis buffer (150 mM NaCl, 50 mM Tris–HCl pH 7.5) supplemented with protease and phosphatase inhibitors and sonicated with UP200 s sonicator (Hielscher Ultrasonics GmbH, Teltow, Germany) for 10 s. Samples were then centrifuged at 10 000 × g for 10 min at 4 °C and the soluble protein fraction (supernatant) was collected and quantified by the Bradford method. A standard curve with known concentrations of bovine serum albumin (BSA, 0.125 to 2 μg/μL) was used to determine protein concentration. Samples were stored at −30 °C until use. Equal amounts of protein (10–40 μg) from each extract were prepared for analysis by western blotting adding to the samples loading buffer at a final concentration of 1x, which were then boiled for 5 min at 95 °C. Proteins were then resolved on 6%, 10% or 15% w/v SDS polyacrylamide gel and subsequently, proteins were transferred to nitrocellulose membranes (Cytiva, Marlborough, MA, USA). Membranes were incubated with primary antibodies at a dilution of 1:1000, either overnight at 4ºC or for 2 h at room temperature. Membranes were then washed and incubated with a secondary mouse IgG Horseradish Peroxidase-conjugated antibody, diluted 1:10 000. Chemiluminescence detection was performed using the Pierce ECL Plus Western Blotting Substrate and the Amersham Imager 680 blot and gel imager (Cytiva, Marlborough, MA, USA). The integrated intensity of each band was calculated using computer-assisted densitometry analysis with ImageJ software (Rasband, W.S., ImageJ, U. S. National Institutes of Health, Bethesda, Maryland, USA, https://imagej.net/ij/, 1997–2018). These values were normalized to the loading control (GAPDH).

### Fluorescence Microscopy and Immunocytochemistry

Fluorescence microscopy and immunocytochemistry were performed as previously described [8]. Before fixing the cells or using them for live-cell microscopy, cells were incubated with 75 nM of MitoView™ Fix 640 for at least 2 h at 37 °C in 5% CO_2_ atmosphere. Cells were fixed in ice-cold methanol for 15 min and blocked with 1% (w/v) BSA in PBST (PBS supplemented with 0.1% Tween-20) for 1 h at RT. Samples were incubated overnight at 4 °C with anti-Nestin, anti-vimentin or anti-S100B, primary antibodies (1:200 in blocking solution). Cells were incubated with the corresponding goat anti-mouse or anti-rabbit secondary antibodies conjugated with AlexaFluor 488, AlexaFluor 546 or AlexaFluor 647 (1:800) for 1 h at RT, and counterstained with the nuclear marker Hoechst 33,342. Images were captured using Leica TCS SPE and Leica DMI6000B systems and the Leica LAS X Core software (Wetzlar, Germany). These systems were equipped with a Leica DFC 365 FX camera and a monochrome CMOS camera (Wetzlar, Germany), respectively. A 63X/1.4 oil objective and a 40X/1.10 water objective (Wetzlar, Germany) were used for imaging, respectively. Subsequent image processing was carried out using ImageJ software.

### Image Analysis

Morphological analysis of nestin distribution within cells was performed using ImageJ. The nestin channel was processed using the “Threshold” function, and subsequent measurements of circularity (using the formula [4 × surface area/perimeter^2^] where 1 indicates a perfect circle, while values approaching 0 suggest increasingly elongated shapes [29]) and area occupied by nestin were obtained. Fluorescently labelled mitochondria were analysed using the “Mitochondria Analyzer” plugin (version 2.1.0) for ImageJ. For each experiment, the mitochondrial channel was converted to an 8-bit image and cropped to include only one cell (Supplementary Fig. 1a,c). The “Adaptive Threshold” method was applied with a block size of 1.55 µm. The optimal C value was empirically determined for each experiment and images were converted into binary cell (Supplementary Fig. 1b,d). From those binary images, several mitochondrial parameters were measured, including the aspect ratio (ratio between the length and width of mitochondrion) [29] giving information about the shape of each mitochondrion and the total branch length (per area) indicating mitochondrial network complexity. Mitochondrial networks were classified into “fragmented” (characterized by small, round mitochondria with minimal branching) and “filamentous” (characterized by extensive branching and long, interconnected mitochondria) [30].

### Quantitative Reverse Transcription Polymerase Chain Reaction (qRT-PCR)

Total RNA was extracted from C6 and C6^Sacs−/−^ cells by means of TRIzol reagent in accordance with the manufacturer’s instructions and quantified by means of NanoDrop™ One (Thermo Fisher Scientific Inc., Waltham, MA, USA). The RNA samples (1 μg) were transformed into cDNA by PCR with NZY Reverse Transcriptase (NZYTech), following manufacturer´s instructions. Quantitative PCR of cDNA samples was made with SensiFAST™ SYBR® Hi-ROX Kit (bio-92020; Bioline) and the corresponding primers (Table 1) in a QuantStudioTM 7 Flex Real-Time PCR System (Thermo Fisher Scientific Inc.). The PCR program was run with an initial denaturation at 95 °C for 2 min, followed by 40 cycles of 5 s at 95 °C and 30 s at 60 °C. A melting curve analysis from 60 °C to 95 °C was conducted to verify amplification specificity. Gene expression levels were normalized to the housekeeping gene hypoxanthine phosphoribosyltransferase (HPRT) and calculated as fold change relative to the control group using the calibration curve method. Statistical analysis was performed using ΔCt values.

**Table 1:** List of the primers used in quantitative PCR

Gene	Primer forward (5’−3’)	Primer reverse (5’−3’)
*HPRT*	GGTGAAAAGGACCTCTCGAAGTG	ATAGTCAAGGGCATATCCAACAACA
*S100B*	CAGGAGCCTCCGGGATGT	TCCTGCTCTTTGATTTCCTCCA
*DRP1*	CGTAGTGGGAACTCAGAGCA	ACCCCATTCTTCTGCTTCAACT
*MFN2*	CAGAGCAGAGCCAAACTGCT	AACATGTTGAGTTCGCTGTCC
*OPA1*	AGAAGTTTCTGAGGCCCTCCTTCTCTT	TTCTTTGTCTGACACCTTCCTGT
*TFAM*	GATGAGTCAGCTCAGGGGAAAT	GGATGAGATCACTTCGCCCA
*LDH*	CCTGTGTGGAGTGGTGTGAA	CCAAGTCTGCCACAGAGAGG

### Flow Cytometry

Cells were washed with PBS, trypsinized and collected to 1.5 ml tubes. After centrifugation at 500 × g for 5 min at room temperature, cells were resuspended in DMEM only with JC-1 dye at a final concentration of 0.5 µM and incubated for 10 min. Then, cells were washed twice with PBS and 10,000 cells were analysed using BD Accuri C6 Plus and CytoFLEX V0-B4-R2 Flow Cytometers in the green (FITC-FL1) and red (PE-FL2) channels. Data were analysed using the Floreada online tool (https://floreada.io/). JC-1 is a ratiometric dye that shows red fluorescence in healthy mitochondria (normal-high mitochondrial potential) and green fluorescence in damaged cells (low mitochondrial potential). Final results are shown as the ratio between FL2- and FL1-positive cells.

### Statistical Analysis

Statistical analysis and graphical representation of data were conducted using GraphPad Prism software (Version 8, GraphPad, San Diego, CA, USA). Data are presented as mean ± standard error of the mean (SEM) from at least three independent experiments. Outliers were removed using ROUT method with Q set to 1%. Statistical comparisons between two experimental groups were made using Student’s t-test. For experiments with more than two groups, data were analysed by means of a one-way ANOVA followed by a Tukey’s multiple comparisons test, with a single pooled variance. Results were considered statistically significant at *p* < 0.05.

## Results and Discussion

### Sacsin Deletion Affects S100B Expression and its Subcellular Distribution

**Fig. 1:**
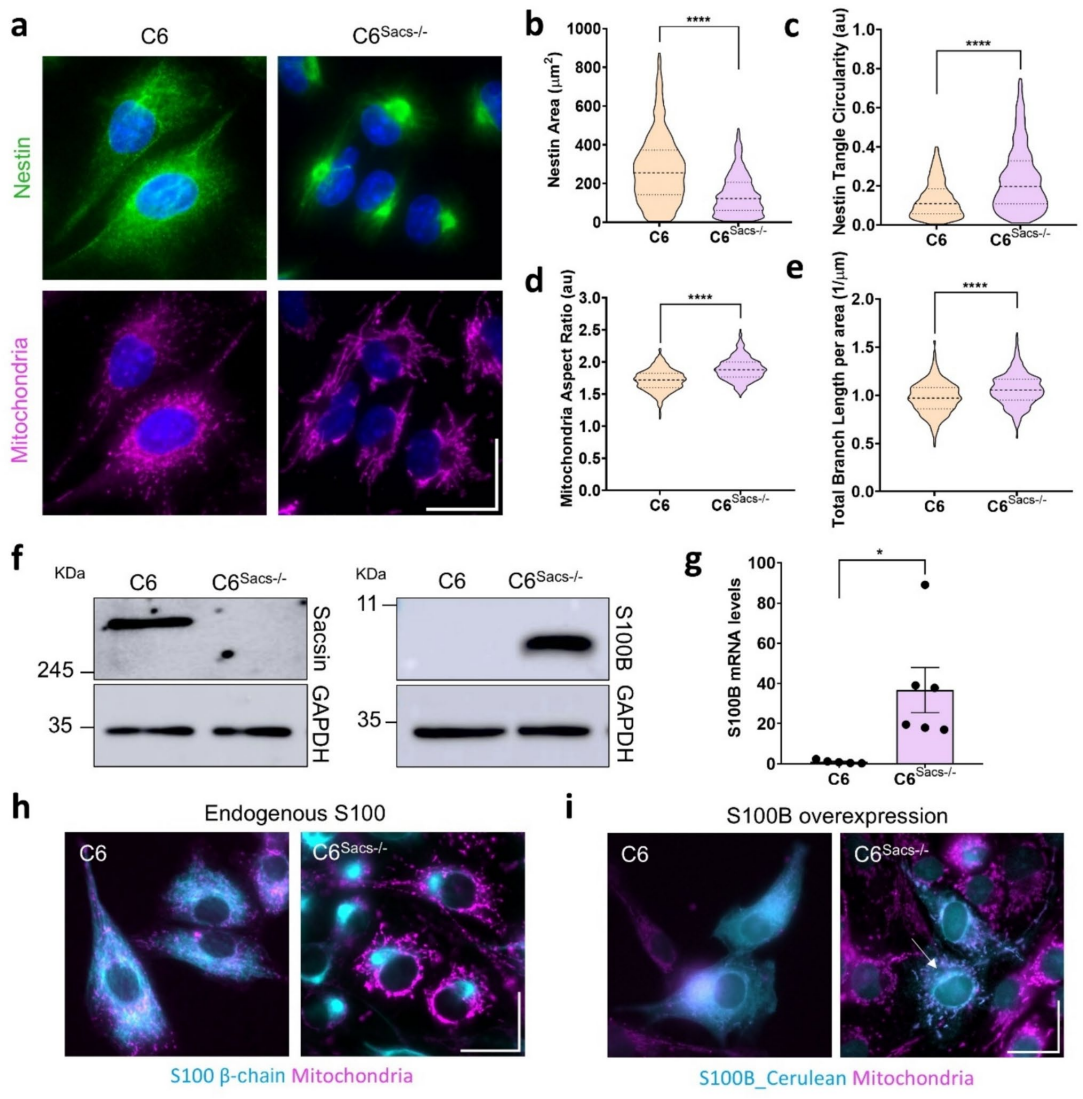
Sacsin deletion increases S100B levels and alters its subcellular distribution. **a** Representative images of C6 and C6^Sacs−/−^ cells showing nestin juxtanuclear accumulation (green), nucleus (blue) and mitochondria (magenta). Scale bar – 20 µm. **b, c** Quantitative analysis of nestin area and nestin tangles circularity, respectively. Data were collected from 6 independent experiments with a total number of 982 WT cells and 1449 KO cells analysed. Data are shown as mean ± SEM; unpaired student’s t-test: *P < 0.05, **P < 0.01, ****P < 0.0001. **d, e** Quantitative analysis of mitochondria aspect ratio (**d**) and total branch length per area (µm) (**e**), respectively. Data were collected from 8 independent experiments with a total number of 481 WT cells and 631 KO cells analysed. Data are shown as mean ± SEM; unpaired student’s t-test: *P < 0.05, **P < 0.01, ****P < 0.0001. **f** Representative western blots showing sacsin deletion and S100B overexpression levels in C6 and C6^Sacs−/−^cells. **g** Levels of S100B mRNA are upregulated in C6^Sacs−/−^ (*n* = 6, *p*-value = 0.0183). Data are expressed as fold change over S100B levels in reference C6 cells. Data are represented as mean values ± SEM and statistical analysis was performed through Student’s t-test. *p < 0.05. **h, i** Representative images of C6 and C6^Sacs−/−^ cells with endogenous S100B (**h**) and overexpression of an exogenous S100B-cerulean construct (**i**), respectively. When overexpressed in the C6^Sacs−/−^cells, S100B locates between IFs tangles and nucleus in C6^Sacs−/−^cells, indicated by white arrow. Scale bar – 20 µm

Alterations of intermediate filaments is a hallmark of all cellular models of ARSACS, including the astroglial (C6 rat glioblastoma cells) [8] and microglia (human HMC3 cells) [31] models recently developed in our laboratory. In the astroglial C6 model of ARSACS, a juxtanuclear accumulation of glial IFs GFAP, Vimentin and Nestin is observed upon sacsin knockout [8]. Nestin tangles were found in a higher percentage of cells (~ 60%) and therefore used as a reliable indicator of IF alterations in subsequent experiments (Fig. 1a). Morphological analyses showed that nestin accumulation in C6^Sacs−/−^ cells could be characterized by a reduction in the cytoplasmic area occupied by nestin and by an increase in the Circularity and Aspect Ratio of nestin tangles (Fig. 1b,c). Both parameters indicate that the shape of nestin distribution is closer to circles or ellipses in C6^Sacs−/−^ cells than their wild type counterparts; and higher Circularity values also indicate that their boundaries are less irregular.

Alterations in the mitochondrial network are also visible in C6^Sacs−/−^ cells, including a depletion of mitochondria in the juxtanuclear region where IFs accumulate (Fig. 1a), in agreement with previous observations [8]. Morphological quantification of these alterations revealed an increase in the mitochondrial aspect ratio and total branch length(normalized per area) in C6^Sacs−/−^ cells (Fig. 1d, e; Supplementary Fig. 1c,d), suggesting the presence of more elongated mitochondria and more intricate mitochondrial networks, respectively.

We next investigated whether sacsin loss impacts the expression levels of S100B. Astrocytes produce and secrete high levels of S100B, especially during neuroinflammation [16, 19, 32]. C6 cells did not express detectable S100B levels in basal conditions (Fig. 1f), consistent with previous studies [33]. However, we observed significant upregulation of S100B upon sacsin deletion at both protein (Fig. 1f) and mRNA levels (Fig. 1g). S100B upregulation was not found in HMC3^Sacs−/−^ human microglial cells (Data not shown), indicating that it could be specific of the astroglial lineage. To evaluate whether the observed increase in S100B expression corresponds to a functional complementation response triggered by sacsin loss, we examined whether the cellular distribution of S100B correlates with the IF aggregates in C6^Sacs−/−^ cells. Endogenous S100B, distributes homogenously throughout the cytoplasm of reference C6 cells (Fig. 1h, left panel), with partial co-localization with mitochondria. In C6^Sacs−/−^ cells, in contrast, S100B accumulates in the same juxtanuclear region as the intermediate filaments (Fig. 1h, right panel). S100B co-localizes with vimentin in both reference and sacsin-knockout C6 cells (Supplementary Fig. 2), confirming previous reports [20, 21]. Expression of exogenous S100B in C6 cells also shows homogeneous distribution of S100B throughout the cytoplasm, having partial mitochondrial and nuclear localization (Fig. 1i, left panel). In contrast, C6^Sacs−/−^ cells exhibited higher S100B signal in the perinuclear region, and accumulating between the nucleus and IF aggregates (Fig. 1i, right panel). This observation suggests that, unlike the homogeneous cytoplasmic distribution observed in wild-type cells, S100B is predominantly retained within (endogenous S100B) or around (exogenous S100B) the hotspots of IF aggregation in C6^Sacs−/−^ cells. This localization aligns with the juxtanuclear distribution of nestin tangles and mirrors the observed colocalization of the HSP70 chaperone with vimentin aggregates in ARSACS patient cells [9] collectively supporting a role for S100B as a chaperone regulating the disrupted dynamics of cellular filaments caused by sacsin deletion.

The overlap of S100B with mitochondria in C6^Sacs−/−^ cells (Fig. 1i, right panel) resembles sacsin mitochondrial association during chaperone-assisted selective mitophagy following mitochondrial damage. S100B is known to interact with mitochondria and mitochondrial proteins such as ATAD3 A [25, 34]. Notably, S100B prevents aggregation of a truncated ATAD3 A mutant, which remains cytoplasmic due to impaired mitochondrial import, and restores its mitochondrial localization [25]. However, mitochondrial ATAD3 A protein was not detected in either C6 or C6^Sacs−/−^ cells (Supplementary Fig. 3). Thus, the colocalization of S100B with mitochondria in C6^Sacs−/−^ cells likely reflects functions that, akin to those of sacsin, remain incompletely understood.

Overall, these results reveal that sacsin deletion induces S100B overexpression and alters its subcellular distribution, with S100B accumulating in juxtanuclear regions and associating with IF aggregates and mitochondria. This suggests a compensatory role for S100B in managing the disrupted cytoskeletal and mitochondrial dynamics caused by sacsin loss, highlighting its potential involvement in cellular adaptations to ARSACS pathology.

### S100B Reverts Pathological Hallmarks in the ARSACS C6 Cell Model

**Fig. 2:**
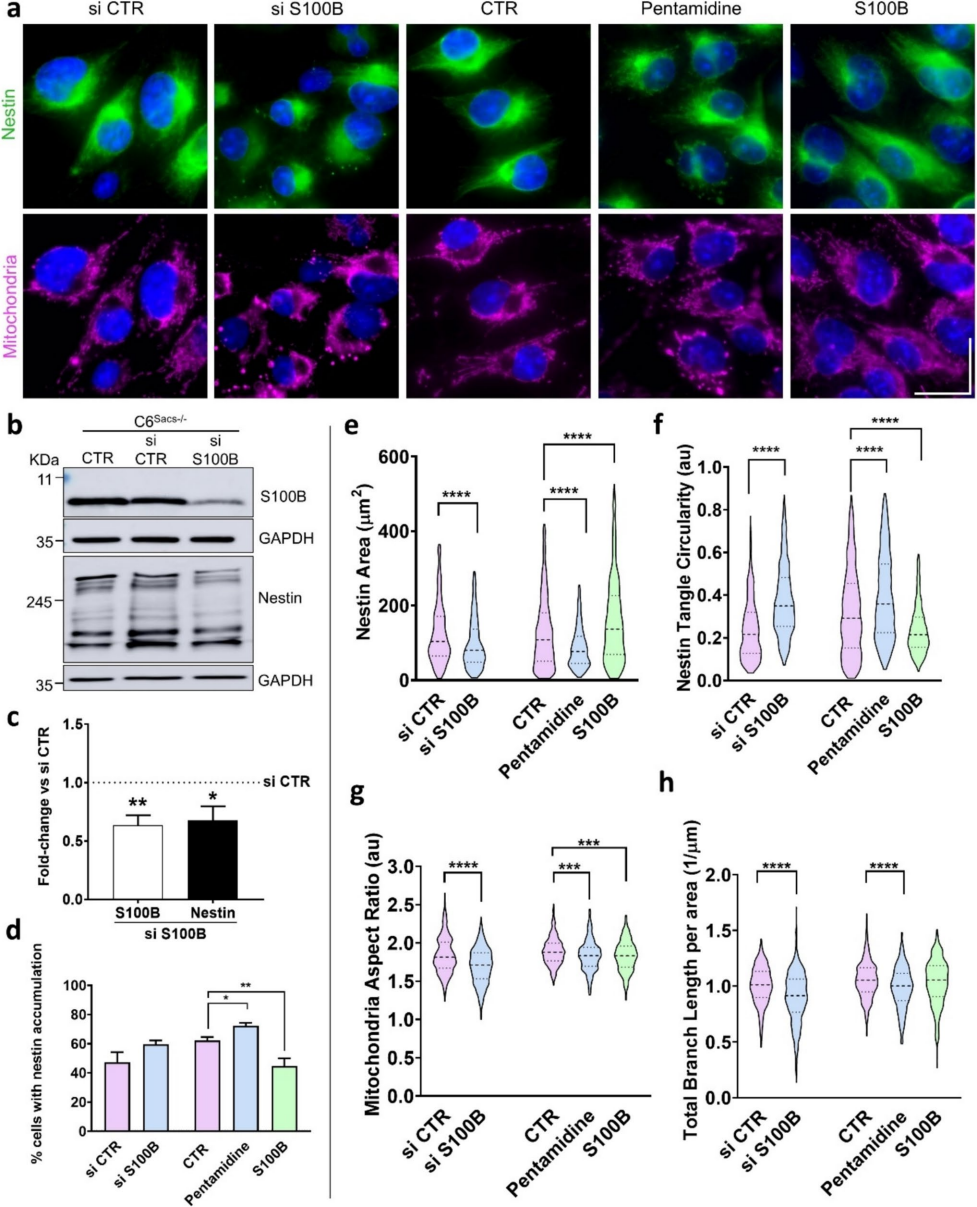
Effects of S100B modulation on nestin accumulation and mitochondrial morphology in C6^Sacs−/−^ cells. **a** Immunocytochemistry images showing nestin distribution (green), mitochondrial network (magenta) and nucleus (blue) after transfection with siRNA control or against S100B; after 48 h incubation with pentamidine (0.5 µM) and after incubation with exogenous S100B (30 µM). Scale bar – 20 µm. **b** Representative western blot showing inhibition of S100B with siRNA and decrease in nestin levels of expression. **c** Densitometric analysis of western blots normalized to GAPDH levels. Fold-change representation of S100B and Nestin comparing to C6^Sacs−/−^ cells transfected with siRNA control (*p*-values = 0.0010 and 0.0284, respectively; unpaired t-test, *n* = 5). **d** Graphical representation of the percentage of cells with nestin accumulation. Data are shown as mean ± SEM; unpaired student’s t-test: *P < 0.05, **P < 0.01. **e, f** Quantitative analysis of nestin area (µm^2^) and nestin tangle circularity, respectively, following S100B inhibition with siRNA (752 cells) or 0.5 µM pentamidine (1422 cells) or exposure to 30 µM of exogenous S100B (1055 cells). siRNA control (628 cells) was used for comparison with siRNA S100B and non-treated C6^Sacs−/−^ cells (2117 cells) were used as control for pentamidine and exogenous S100B. Data were obtained from at least 3 independent experiments and are shown as mean ± SEM; one-way ANOVA followed by Tukey’s test: *P < 0.05, **P < 0.01, ****P < 0.0001. **g, h** Quantitative analysis of mitochondria aspect ratio and total branch length per area (1/µm), respectively following S100B inhibition with siRNA (736 cells) or 0.5 µM pentamidine (428 cells) or exposure to 30 µM of exogenous S100B (398 cells). siRNA control (498 cells) was used for comparison with siRNA S100B and non-treated C6^Sacs−/−^ cells (636 cells) were used as control for pentamidine and exogenous S100B. Data were collected from at least 3 independent experiments. Data are shown as mean ± SEM; one-way ANOVA followed by Tukey’s test: *P < 0.05, **P < 0.01, ****P < 0.0001

To determine whether sacsin deletion phenotypes can be rescued by S100B expression in the ARSACS C6 model, we designed experiments to manipulate S100B functional levels in C6^Sacs−/−^ cells. Specifically, we conducted experiments to either inhibit or increase S100B expression (Fig. 2). For inhibition, we used small interfering RNA (siRNA) to knock down S100B expression, while functional pharmacological inhibition was achieved using the inhibitor pentamidine. To increase S100B levels, we supplemented the cell cultures with exogenous purified recombinant S100B, as previous studies have established that this approach is non-toxic and facilitates S100B uptake by cells [35]. Inhibition of S100B expression using siRNA (*p*-value = 0.0010) led to a reduced expression of nestin (*p*-value = 0.0284) (Fig. 2b, c), and a slight, non-significant increase in the proportion of cells with nestin tangles (Fig. 2d). Additionally, the area occupied by nestin was reduced and the Circularity of nestin aggregates was increased (Fig. 2e, f), suggesting more concentrated juxtanuclear aggregation of IFs upon S100B knockdown. A further disruption of mitochondrial network was also observed upon S100B knockdown in C6^Sacs−/−^ (Fig. 2a), with a decrease in the aspect ratio and total branch length, indicating more fragmented mitochondria (Fig. 2g, h). These results indicated that abrogation of S100B exacerbates the IF and mitochondrial phenotypes characteristic of ARSACS cells.

Pharmacological S100B inhibition by pentamidine [36] largely confirmed these results. Pentamidine (0.5 µM) increased the percentage of cells with nestin tangles (Fig. 2a, d) and turned nestin tangles smaller and more circular (Fig. 2a, e, f). Additionally, the mitochondrial network became more fragmented, reducing aspect ratio and total branch length per area (Fig. 2a, g, h), upon incubation with the drug. This is consistent with previous studies in prostate cancer cells, where pentamidine also induced mitochondrial morphological changes [27]. Conversely, supplementation of cells with exogenous recombinant S100B protein (30 µM) led to lower percentage of cells with nestin tangles (Fig. 2d), a more widespread distribution of nestin (Fig. 2a), and a reduction in circularity of nestin tangles (Fig. 2e, f); but did not revert mitochondrial alterations in C6^Sacs−/−^ cells (g, h). It is possible that endogenous S100B is saturating the mechanisms involved in preservation of mitochondrial morphology, and addition of exogenous S100B therefore cannot improve further this parameter. These results support the role of S100B as a chaperone and are consistent with previous studies where S100B modulates assembly and disassembly of type III IFs and microtubules [22, 32, 37].

### S100B Upregulation Prevents ARSACS-Related Alterations in Mitochondrial Homeostasis but not in Mitochondrial Function

**Fig. 3:**
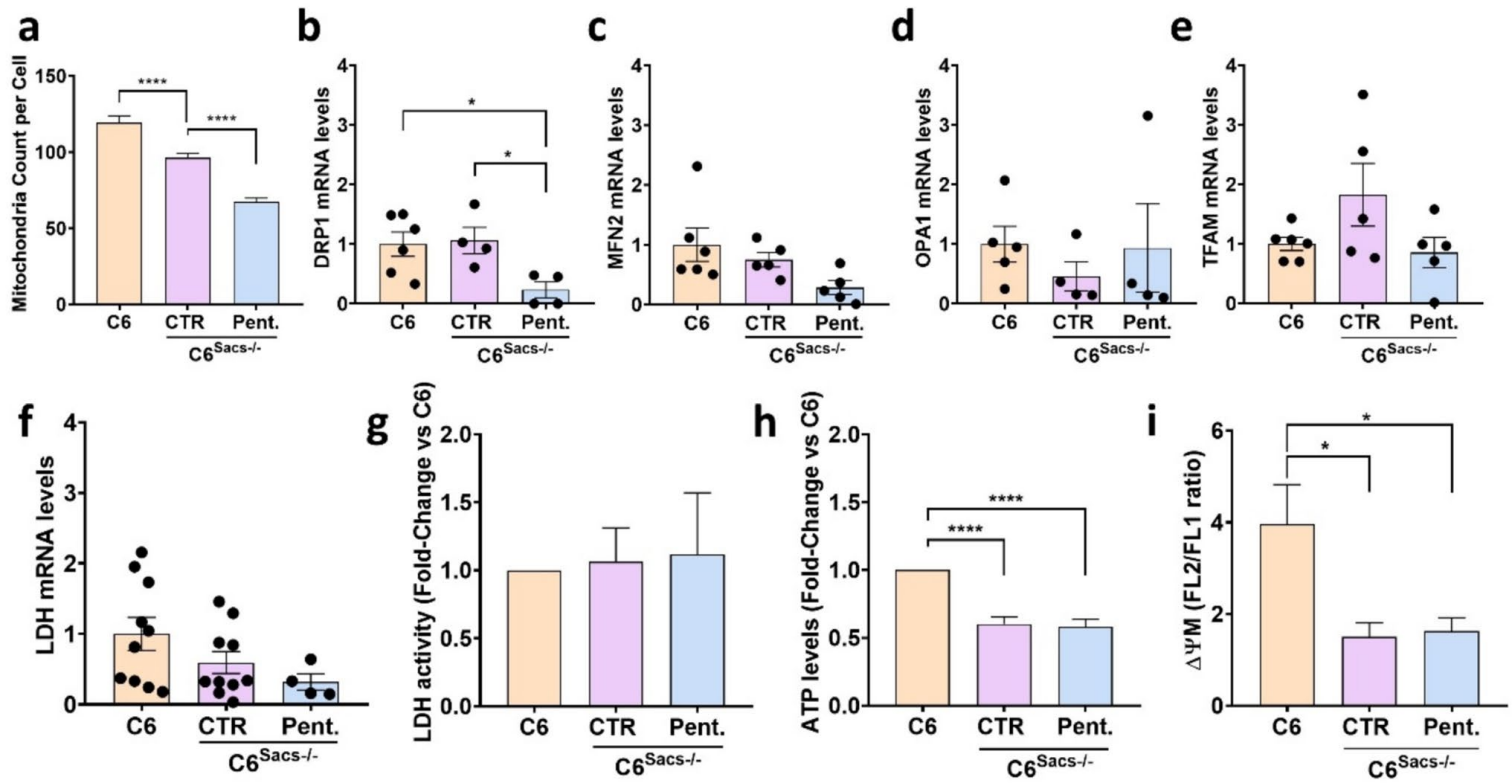
Pharmacological inhibition of S100B activity influences mitochondrial homeostasis and function. **a** Number of mitochondria per cell comparing C6 (483 cells), C6^Sacs−/−^ (636 cells) and C6^Sacs−/−^ treated with pentamidine (428 cells). Data are shown as mean ± SEM; one-way ANOVA followed by Tukey’s test: ****P < 0.0001. **b-f** Transcript levels of DRP1, MFN2, OPA1, TFAM and LDH in C6 and C6^Sacs−/−^ incubated with pentamidine or the vehicle (DMSO 0.1% v/v). Data are represented as mean ± SEM (*n* = 4–10 independent experiments), normalized versus reference C6 cells. Statistical analysis was performed through one-way ANOVA followed by Tukey’s test: *P < 0.05. **g** LDH activity in the same experimental groups. Data are represented as mean ± SEM of 7 independent experiments, normalized versus reference C6 cells. **h** ATP levels are represented as mean ± SEM of 5 independent experiments, normalized versus reference C6 cells. Statistical analysis was performed through one-way ANOVA followed by Tukey’s test:****p < 0.0001. **i** Graphical representation of ratio between JC-1 aggregates (FL2—585/42 nm) and JC-1 monomers (FL1—530/30 nm). Data are represented as mean ± SEM (*n* = 4 independent experiments), Statistical analysis was performed through one-way ANOVA followed by Tukey’s test:*p < 0.05

To understand better the role of S100B on the mitochondrial alterations caused by sacsin loss, we analysed a series of parameters related to mitochondrial homeostasis and function. Sacsin deletion decreased significantly the number of mitochondria per cell (Fig. 3a), and pentamidine potentiated this effect, suggesting that S100B upregulation partially compensates for the effects of sacsin loss on mitochondrial content. Next, we measured the expression levels of the MFN2, DRP1, OPA1 and TFAM genes, involved in mitochondrial fusion and fission (Fig. 3b-e). Sacsin deletion did not impair the expression of any of them, but pentamidine significantly reduced the expression of fission promoter DRP1 in C6^Sacs−/−^ cells. We did not find significant changes in the expression and activity of LDH (Fig. 3f-g), an indicator of a possible shift from mitochondrial-based OXPHOS metabolism towards aerobic glycolysis and fermentative pathways independent from the mitochondrial respiratory chain. Finally, we observed a significant decrease in overall ATP levels and in mitochondrial membrane potential in C6^Sacs−/−^ cells, indicating mitochondrial dysfunction, but these effects were not enhanced by pentamidine (Fig. 3h-i). Altogether, these results suggest that S100B upregulation compensates for sacsin loss-related decrease in overall number of mitochondria and disruption in mitochondrial fission, but not for the alterations in their respiratory and energy-producing functions.

## Conclusion

The results of this study suggest that S100B compensates for sacsin loss in ARSACS cell models, highlighting its neuroprotective role through chaperone activity in preserving IF integrity and mitochondrial morphology. The complementary roles of sacsin and S100B, particularly in their shared involvement in IF and mitochondrial maintenance, point to a coordinated cellular response to proteostasis stress in the central nervous system. Although insufficient to fully rescue the ARSACS phenotype, S100B’s compensatory role may be particularly relevant in cases of partial sacsin depletion, such as mutations in the sacsin J-domain or in compound heterozygous individuals, which are challenging possibilities to investigate in future studies. Altogether, this study provides new insights into the mechanisms involving S100B in ARSACS and other neurodegenerative disorders characterized by IF disruption and proteostasis imbalance and highlights the potential for pharmacological modulation of S100B as a therapeutic strategy.

## Supplementary Information

Below is the link to the electronic supplementary material.

Supplementary file1 (DOCX 1380 KB)

Supplementary file2 (PDF 232 KB)

## Data Availability

No datasets were generated or analysed during the current study.
