# Supplementary material for: S100B Mitigates Cytoskeletal and Mitochondrial Alterations in a Glial Cell Model of Autosomal Recessive Spastic Ataxia of Charlevoix-Saguenay
Source: Mol Neurobiol. 2025 May 19;62(9):12296–306. doi: 10.1007/s12035-025-05057-3 (PMC12367932; doi:10.1007/s12035-025-05057-3)
Supplement: Supplementary file 1 — Supplementary file1 (DOCX 1380 KB) [file 12035_2025_5057_MOESM1_ESM.docx]

# **Supplementary Data**


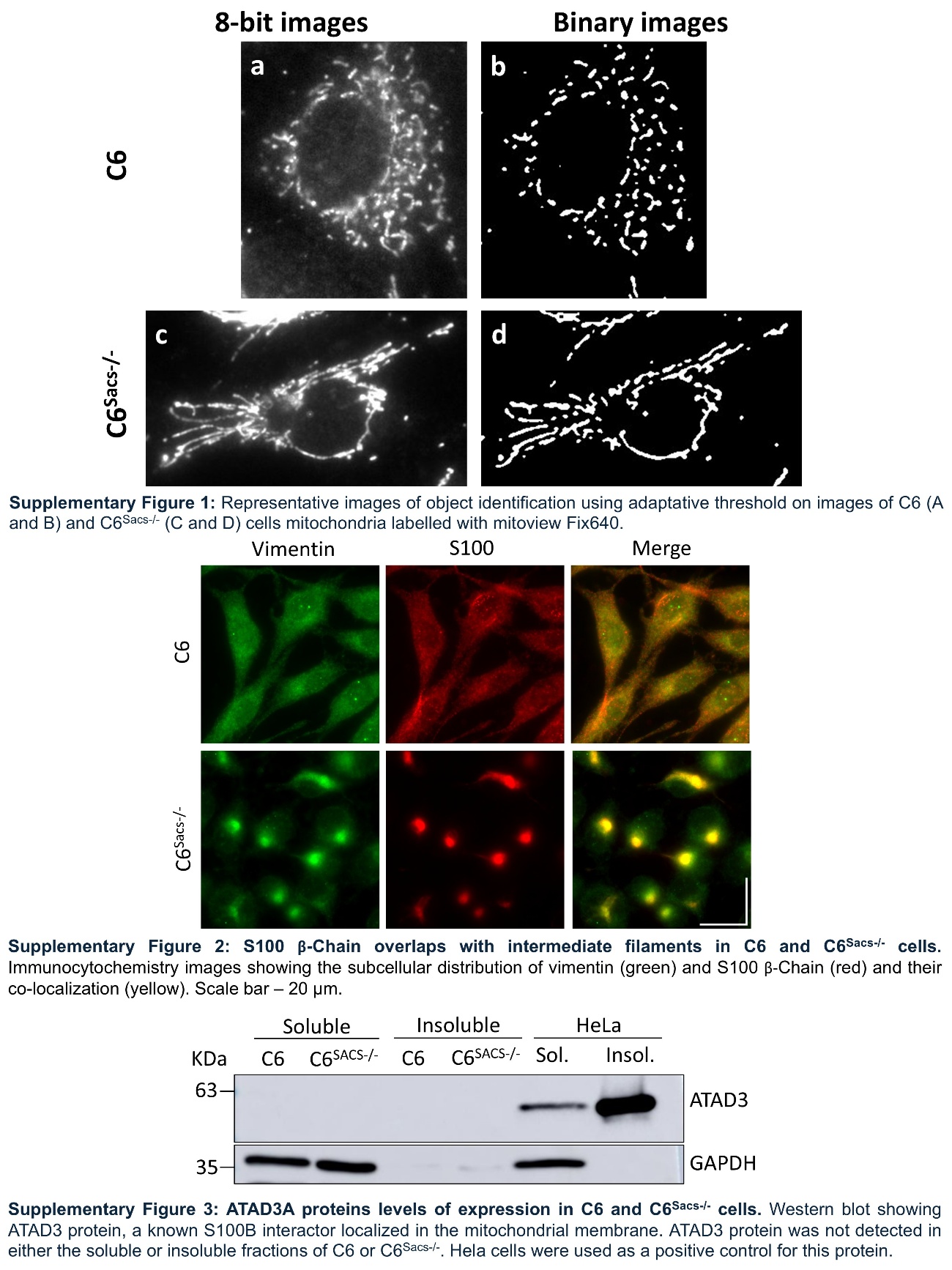


***Supplementary Figure 1:*** *Representative images of object identification using adaptative threshold on images of C6 (a and b) and C6Sacs-/- (c and d) cells mitochondria labelled with Mitoview Fix640****.***


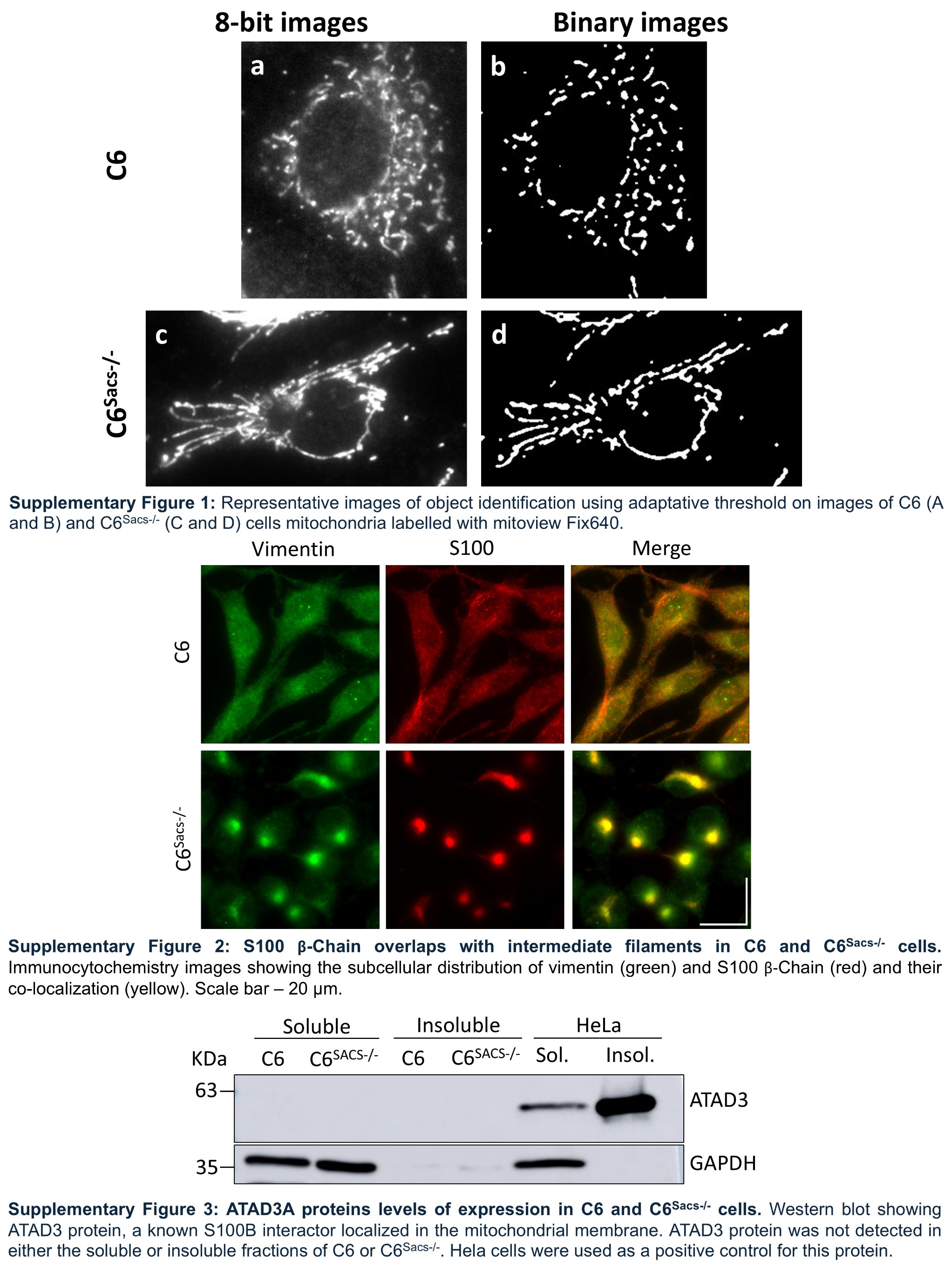


**Supplementary Figure 2: S100 β-Chain overlaps with intermediate filaments in C6 and C6^Sacs-/-^ cells.** Immunocytochemistry images showing the subcellular distribution of vimentin (green) and S100 β-Chain (red) and their co-localization (yellow). Scale bar – 20 µm.


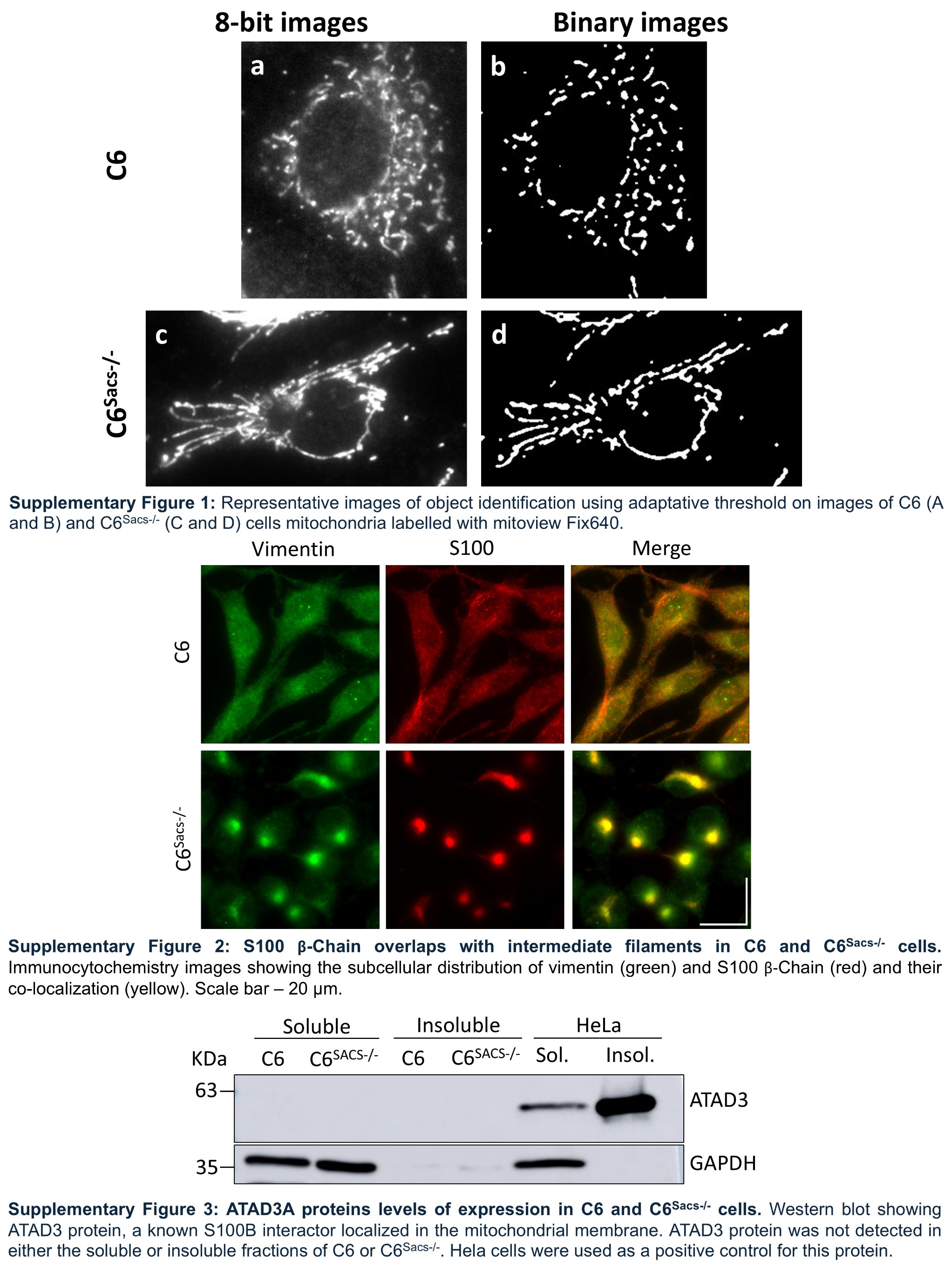


**Supplementary Figure 3: ATAD3A proteins levels of expression in C6 and C6^Sacs-/-^ cells.** Western blot showing ATAD3 protein, a known S100B interactor localized in the mitochondrial membrane. ATAD3 protein was not detected in either the soluble or insoluble fractions of C6 or C6^Sacs-/-^. Hela cells were used as a positive control for this protein.
