# Supplementary material for: S100B Mitigates Cytoskeletal and Mitochondrial Alterations in a Glial Cell Model of Autosomal Recessive Spastic Ataxia of Charlevoix-Saguenay
Source: Mol Neurobiol. 2025 May 19;62(9):12296–306. doi: 10.1007/s12035-025-05057-3 (PMC12367932; doi:10.1007/s12035-025-05057-3)

Supplementary Material – Western Blot Membranes

Figure 1

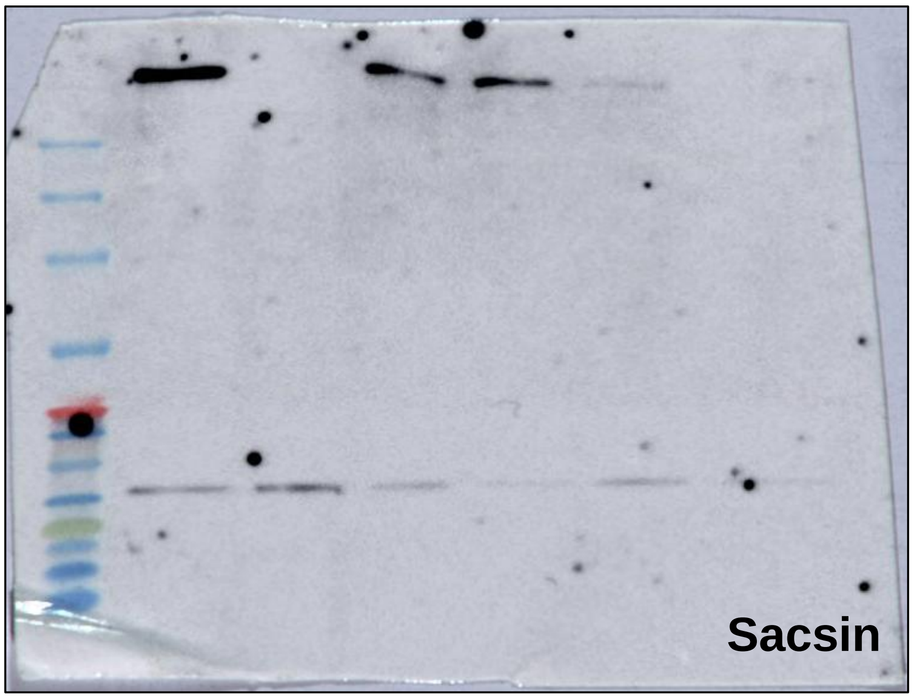

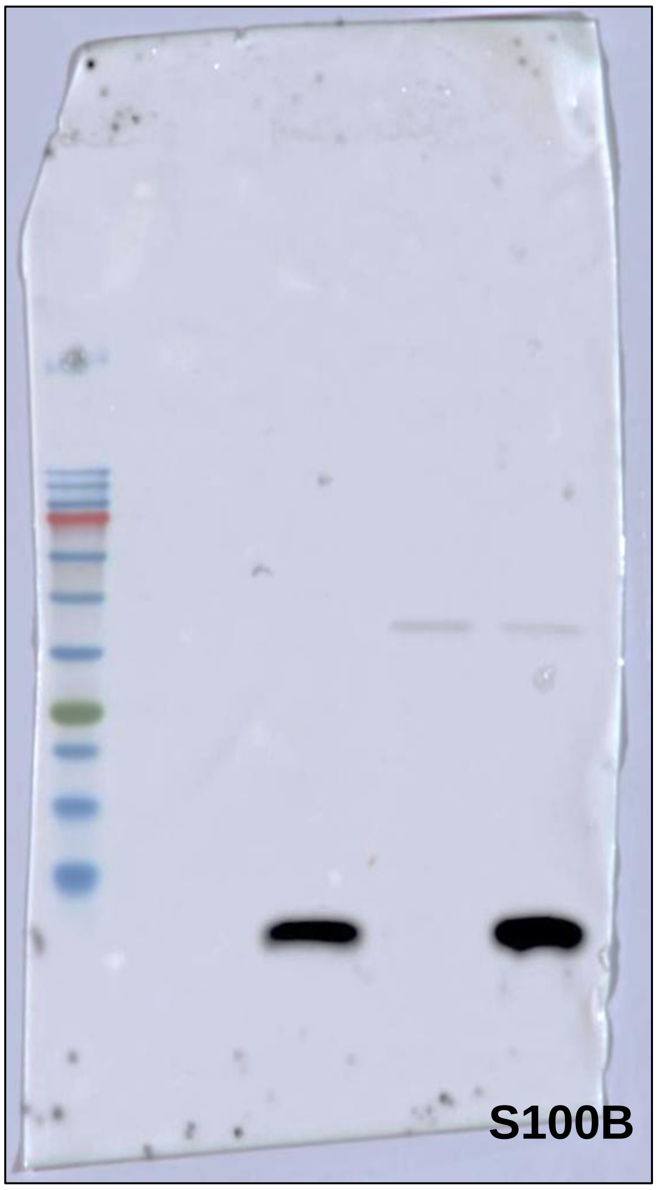

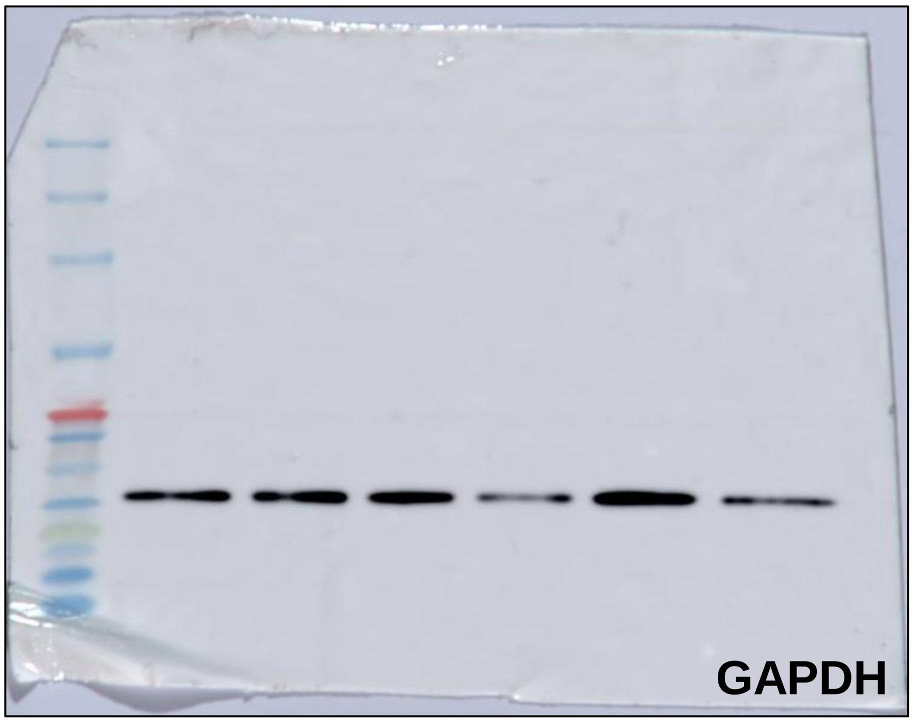

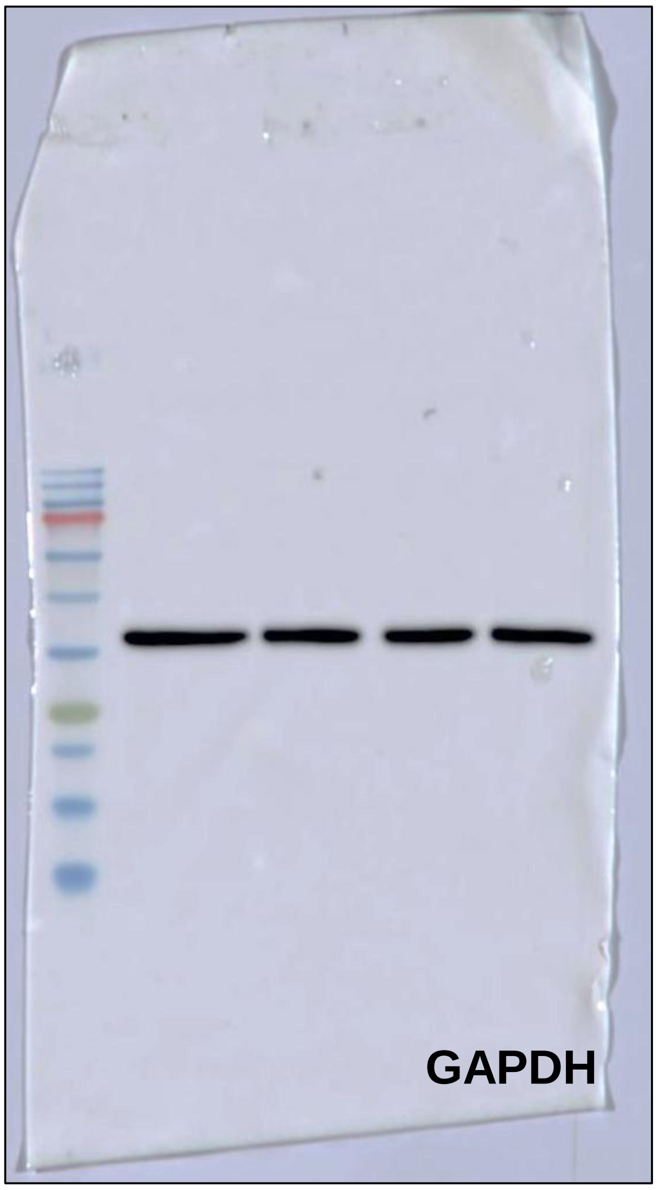

Supplementary Material – Western Blot Membranes

Figure 2

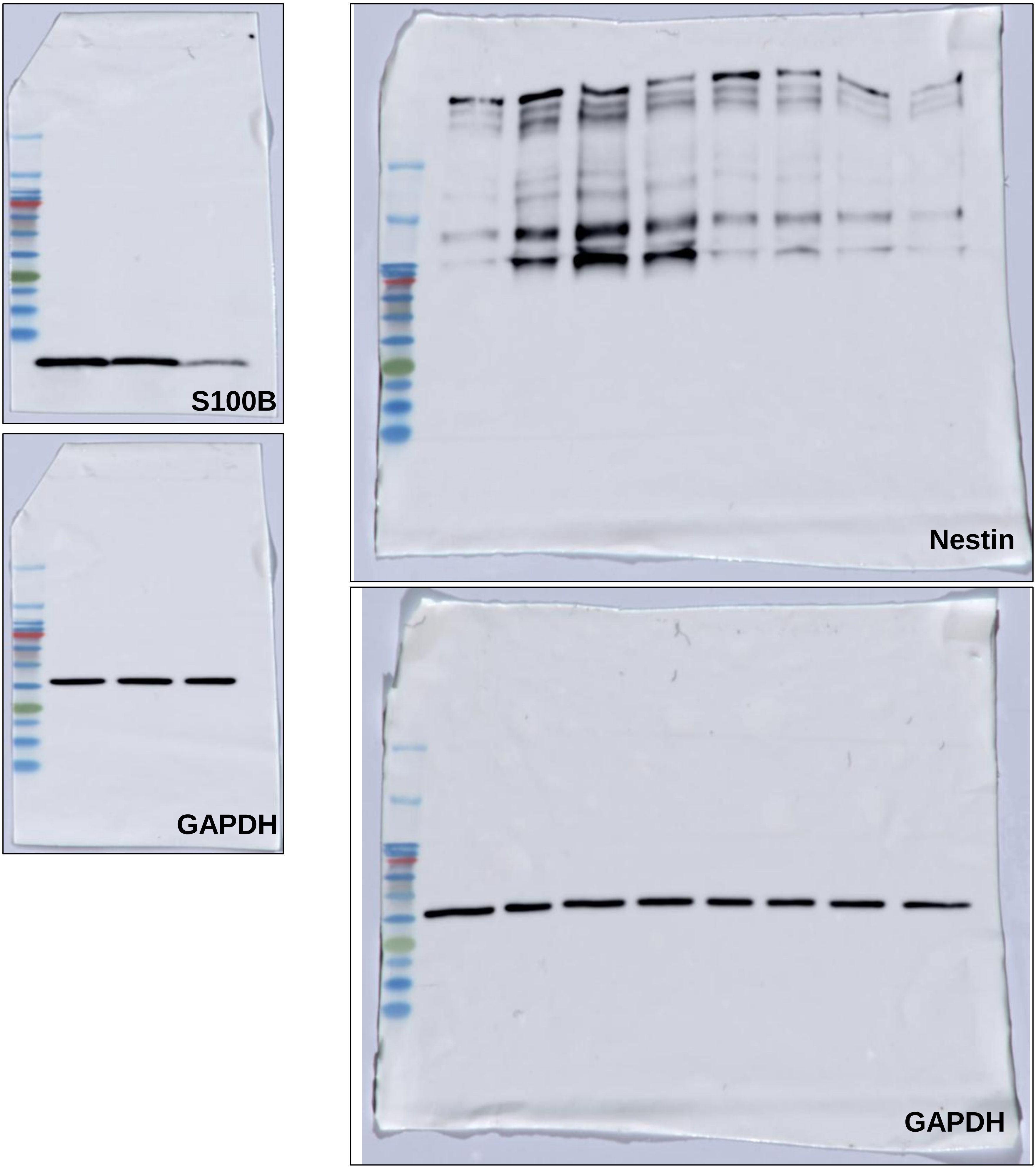

Supplementary Material – Western Blot Membranes

Supplementary Figure 2

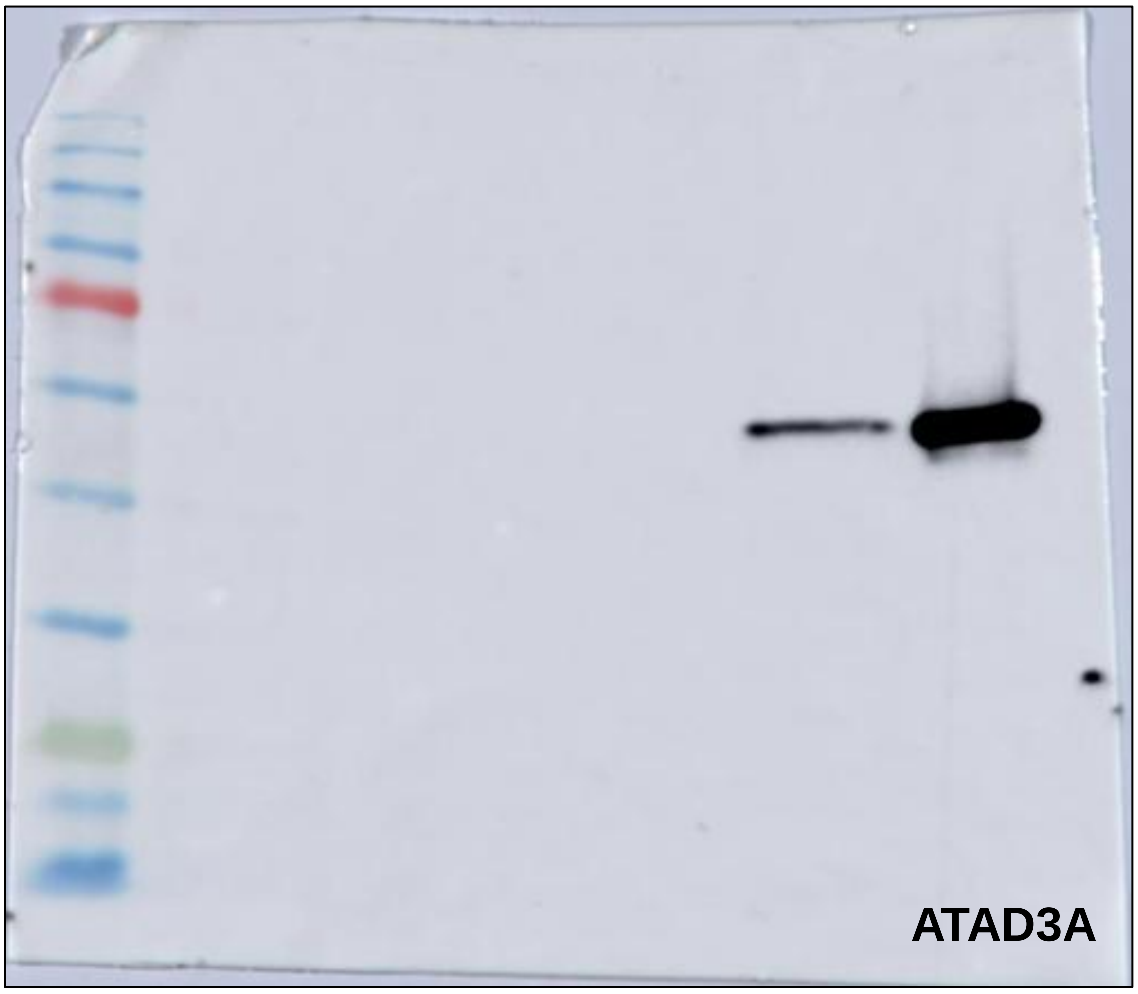

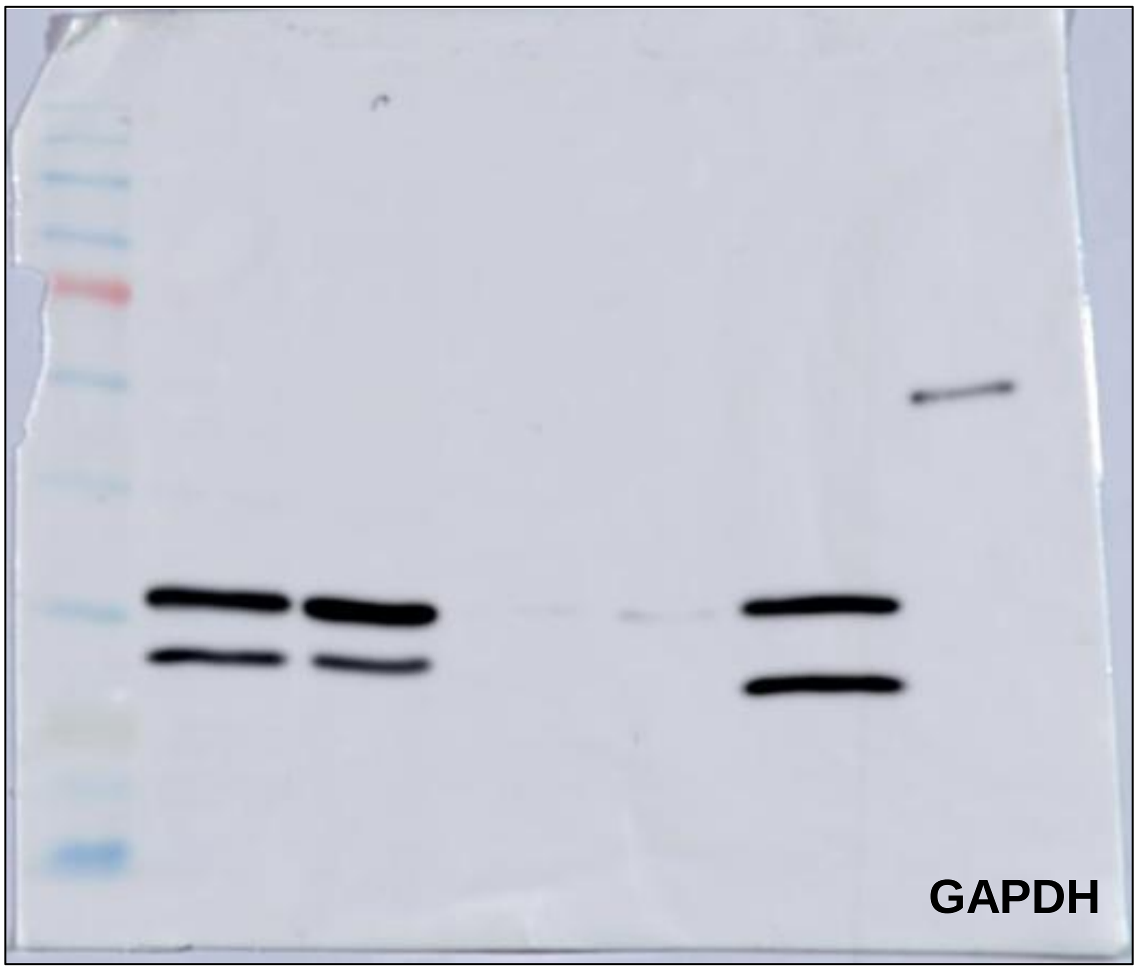

Supplement: Supplementary file 2 — Supplementary file2 (PDF 232 KB) [file 12035_2025_5057_MOESM2_ESM.pdf]
